# The clinical characteristics of focal acute pancreatitis based on imaging diagnosis: comparison with non-localized acute pancreatitis- a preliminary result

**DOI:** 10.1186/s12876-023-03015-8

**Published:** 2023-11-09

**Authors:** Mengmeng Ding, Renren Wang, Huawei Xu, Meng Li, Tao Zhou, Yueyue Li, Yanjing Gao, Xiaomeng Gu

**Affiliations:** 1 Department of Gastroenterology, Qilu Hospital of Shandong University, Wenhua Xi Rd, 107, Jinan, Shandong 250012 People’s Republic of China; 2 Department of Geriatric Medicine, Qilu Hospital of Shandong University, Wenhua Xi Rd, 107, Jinan, Shandong 250012 People’s Republic of China

**Keywords:** Focal acute Pancreatitis, Non-localized acute Pancreatitis, Clinical features

## Abstract

**Background:**

Focal acute pancreatitis is a special type of acute pancreatitis, which diagnosis is based on image showing a focal mass formation in the pancreas. For acute pancreatitis with or without focal inflammatory enlargement, little is known on differences between them. Our purpose was to find differences between focal acute pancreatitis and non-localized acute pancreatitis.

**Methods:**

We reviewed the medical records of a total of 24 patients diagnosed with focal acute pancreatitis by imaging and clinical diagnosis, and 27 cases of acute pancreatitis which manifest non-localized pancreas inflammation were selected as the control group. The differences of the two groups were compared to describe their clinical characteristics.

**Results:**

Differences in bloating (4.2% VS 29.6%,*P* = 0.026), abdominal tenderness (58.3% VS 85.2%,*P* = 0.032), peripheral blood neutrophil ratio (60.1 ± 23.3VS 75.9 ± 12.6,*P* = 0.004), serum D-Dimer (0.40(0.25,0.98) VS 1.59(0.49,4.63),*P* = 0.008), serum GGT (40(25,91) VS120(22,383),*P* = 0.046), serum amylase(435(241,718) VS 591(394,1333),*P* = 0.044) and lipase(988(648,1067) VS 1686(525,2675),*P* = 0.027) between focal acute pancreatitis and non-localized acute pancreatitis groups were statistically significant. However, difference of the severity of two groups was not statistically significant (*P* = 1.000).

**Conclusion:**

Compared with non-localized acute pancreatitis, changes in symptoms, signs and laboratory indicators of focal acute pancreatitis are non-obvious, however, there was no significant difference in the severity of two groups, indicating that we should pay more attention to diagnosis of focal acute pancreatitis in clinical practice.

## Background

Focal pancreatitis(FP) is a special type of pancreatitis, which presenting as a local inflammatory mass of pancreas in imaging. FP is caused by various factors including gallstone diseases, alcohol, trauma, iatrogenic injury, autoimmune factors, tumors [1–5]. FP can be clinically asymptomatic or present as acute or chronic pancreatitis. There have been lots of research on distinguishing of focal chronic pancreatitis, for it may mimic malignant tumors of the pancreas including pancreatic ductal adenocarcinoma [6–8]. Acute pancreatitis (AP) is one of the most common gastrointestinal condition, characterized by acute inflammatory reactions and cellular damage of pancreas as a result of inappropriate release and activation of trypsinogen to trypsin within the acini [9]. The incidence of AP is (4.9–73.4) / 10 0,000 worldwide and has increased year by year [10, 11], the overall mortality rate of AP is about 5%, which is associated with disease severity, it’s higher when SAP with infection [12, 13]. However, considering timely and accurate diagnosis is particularly important for AP, little is known on focal AP. For differences in etiology composition, clinical manifestations, laboratory tests between focal AP and non-localized AP, as far as we know, there are no relevant reports.

Therefore, the present study aim to find differences between focal AP and non-localized AP, to assist clinicians detect and treat the disease in time.

## Methods

Patients: The study was approved by the Medical Ethics Committee of the Qilu Hospital of Shandong University. A total of 24 patients diagnosed with focal AP in Qilu Hospital of Shandong University between January 1, 2014 and December 31, 2021 were obtained from clinical case database. As a comparison group, a total of 27 AP patients without focal mass forming on imaging were enrolled from the same hospital and period. Inclusion criteria include: (1) patients diagnosed with FP by computed tomography (CT)(CT was done within 48 h of admission. If there was more than one scan, imaging test which is closest to the time of admission was choosed); (2) meet the clinical diagnostic criteria for AP; (3) improved the collection of relevant medical history and laboratory examination within 48 h of imaging examination; (4) after the onset of illness, he or she was hospitalized in Qilu Hospital of Shandong University or had complete and continuous clinical data from other hospitals since the onset of illness. Exclusion criteria include:(1) those with severe mental illness and lack of self-awareness; (2) patients who were automatically discharged from the hospital when their condition did not deteriorate significantly or was not life-threatening; (3) whose medical records were incomplete; (4) autoimmune pancreatitis.

Definitions of symptoms in the study: Abdominal pain-Pain from below the ribs to the part above the groin; Bloating-Subjectively feeling a fullness of part or all of the abdomen; Nausea-Epigastric discomfort and a feeling of urgency to vomit; Vomiting-The contents of the esophagus, stomach, and intestines are forcefully squeezed through the esophagus and spit out of the mouth; Fever-Body temperature rises above the normal range; Diarrhea-Increased droppings volume, hydration, and frequency;Jaundice-Serum bilirubin is too high and deposited in tissues, causing sclera, mucous membranes, and skin to become yellow; Weight loss-Involuntary weight loss of more than 5% of normal weight in 6 months.

Statistical analysis: The Kolmogorov-Smirnov test was used to determine whether the data conform to the normal distribution. The measurement data that conformed to the normal distribution were expressed in the form of mean soil standard deviation, and the independent sample t-test was used for intergroup comparison; the measurement data that did not conform to the normal distribution were expressed as the median and quartile spacing, and the rank sum test was used for intergroup comparison. Categorical variables were expressed as composition ratios or percentages, and intergroup differences were measured by chi-square tests or Fisher’s precise tests. Statistical analyses were performed using IBM SPSS Statistical 26.0. A *P* value < 0.05 was considered statistically significant.

## Results

### Comparison of basic clinical data in patients with focal AP and non-localized AP

A total of 24 patients with focal AP were included, of which 14 were males, accounting for 58.3% of the group, 10 cases were females, accounting for 41.7%, with an average age of (45.83 ± 14.06) years; a total of 27 patients with non-localized AP were included, of which 17 were males, accounting for 63.0%, and 10 cases of women, accounting for 37.0%, with an average age of (44.48 ± 17.95) years. Both groups of patients showed a large proportion of men, but the difference in sex composition ratio was not statistically significant (*P* = 0.735), and the age difference between the two groups was not statistically significant (*P* = 0.768).

Etiology analysis was performed on two groups: in focal AP group were 10 cases of biliary origin (41.7%), 2 cases of hypertriglyceridemia (8.3%), 5 cases of alcohol (20.8%), 5 cases of idiopathic (20.8%), 2 cases of tumor-related factors (8.3%), and 0 case of structurally related factors; in non-localized AP group are 15 cases of biliary origin (55.6%), 5 cases of hypertriglyceridemia (18.5%), 5 cases of alcohol (18.5%), idiopathic (3.7%), 0 case of tumor-related factors, and 1 case of pancreatic division (3.7%). Tumor-related causes occurred in focal AP group, but the etiological composition ratio was not statistically significant (*P* = 0.216) between two groups, and the other etiological composition ratios between two groups were not statistically significant (*P* > 0.05) (Table 1).

**Table 1:** Clinical baseline and etiological composition of patients in groups of focal AP and non-localized AP

	Focal AP	Non-localized AP	*P*
	n = 24	n = 27	
Age(years)	45.83 ± 14.06	44.48 ± 17.95	0.768
Male, n(%) Etiological composition, n(%)	14(58.3%)	17(63.0%)	0.735
Biliary origin	10(41.7%)	15(55.6%)	0.322
Hypertriglyceridemia	2 (8.3%)	5(18.5%)	0.425
Alcohol	5 (20.8%)	5(18.5%)	1.000
Idiopathic	5(20.8%)	1(3.7%)	0.088
Tumor-related factors	2(8.3%)	0(0.0%)	0.216
Structurally related factors	0(0.0%)	1(3.7%)	1.000

### Clinical symptom comparison in patients with focal AP and non-localized AP

In focal AP, the average course of the disease from onset to presentation was (4.71 ± 3.18) days, and the average course of patients in non-localized AP group was (4.74 ± 3.07) days, and the difference was not statistically significant (*P* = 0.971). A total of 0 case (0.0%) progressed to moderately severe acute pancreatitis (MSAP) in the two groups, and 6 cases (11.8%) progressed to severe acute pancreatitis (SAP) in two groups, including 3 cases (12.5%) in focal AP group and 3 cases (11.1%) in non-localized AP group, with no statistically significant difference (*P* = 1.000). There were totally 2 patients were transferred from another hospital,including two patients in focal AP group (8.3%) and zero in non-localized AP (0.0%), with no statistically difference (*P* = 0.216). There were totally 5 patients required ICU admission, including two patients in focal AP group (8.3%) and three in non-localized AP (11.1%), the difference was not statistically significant (P = 1.000).

In this study, all of the two groups of patients presented with abdominal pain of different degrees and different parts, and the symptoms of middle and upper quadrant pain, left upper quadrant pain, right upper quadrant pain, total abdominal pain, and peri-umbilical pain in the focal AP group were 15 cases (62.5%), 2 cases (8.3%), 5 cases (20.8%), 2 cases (20.8%), 2 cases (8.3%), 2 cases (8.3%), 2 cases (8.3%), and 0 case (0.0%), respectively; there were18 cases (66.7%), 1 case (3.7%), 1 case (3.7%), 5 cases (18.5%), 2 cases (7.4%), respectively in the non-localized AP group. However, there was no statistically significant difference in the composition ratio of pain sites between two groups (*P* = 0.180). There were 9 patients (17.6%) with bloating symptoms, including 1 case (4.2%) in focal AP group and 8 cases (29.6%) in non-localized AP group, and the difference in the composition ratio of bloating symptoms between the two groups was statistically significant (*P* = 0.026). Patients presenting with fever, nausea and vomiting, diarrhea, jaundice, and weight loss in focal AP group were 1 (4.2%), 12 (50.0%), 0 (0.0%), 1 (4.2%), and 2 (8.3%), respectively; in non-localized AP group were 3 cases (11.1%), 19 cases (70.4%), 1 case (3.7%), 3 cases (11.1%), and 1 case (3.7%), respectively, and the difference was not statistically significant (*P*>0.05).

Among the signs of the two groups, a total of 37 cases (72.5%) showed abdominal tenderness, including 14 cases (58.3%) in focal AP group and 23 cases (85.2%) in non-localized AP group, the difference was statistically significant (*P* = 0.032).

In this study, there were 13 patients with a history of AP (25.5%), including 4 cases (16.7%) in focal AP group and 9 cases (33.3%) in non-localized AP group, and there was no statistically significant difference between two groups (*P* = 0.173). Zero case (0.0%) with a history of chronic pancreatitis (CP). In this study, patients with diabetes mellitus and gastric diseases were 6 (25.0%) and 3 (12.5%) in focal AP, and 3 (11.1%) and 4 (14.8%) in non-localized AP group, respectively, and the differences were not statistically significant (*P* = 0.276 and *P* = 1.000) (Table 2).

**Table 2:** The main symptoms, signs, and history of patients in groups of focal AP and non-localized AP

	All n=51, n(%)	focal AP n=24, n(%)	non-localized AP n=27, n(%)	*P*	
MSAP/SAP	6(11.8%)	3(12.5%)	3(11.1%)	1.000	
Abdominal pain	51(100.0%)	24(100.0%)	27(100.0%)	0.180	
Bloating	9(17.6%)	1(4.2%)	8(29.6%)	0.026*	
Nausea and vomiting	31(60.8%)	12(50.0%)	19(70.4%)	0.137	
Fever	4(7.8%)	1(4.2%)	3(11.1%)	0.612	
Diarrhea	1(2.0%)	0(0.0%)	1(3.7%)	1.000	
Jaundice	4(7.8%)	1(4.2%)	3(11.1%)	0.612	
Weight loss	3(5.9%)	2(8.3%)	1(3.7%)	0.595	
Abdominal tenderness	37(72.5%)	14(58.3%)	23(85.2%)	0.032*	
History of AP	13(25.5%)	4(16.7%)	9(33.3%)	0.173	
Diabetes mellitus	9(17.6%)	6(25.0%)	3(11.1%)	0.276	
Gastric disease	7(13.7%)	3(12.5%)	4(14.8%)	1.000	

### Laboratory test results of patients in groups of focal AP and non-localized AP

The clinical laboratory test indicators of the two groups of patients are shown in Table 3. The neutrophil ratio, D-dimer, γ-glutamyltransferase, amylase, and lipase levels between the two groups were statistically significant, with p-values of 0.004, 0.008, 0.046, 0.044, and 0.027, respectively. There were no significant differences in the remaining laboratory test indicators between the two groups (*P* > 0.05) (Table 3).

**Table 3:** Laboratory test results of patients in groups of focal AP and non-localized AP

	focal AP	non-localized AP	*P*
	n = 24	n = 27	
WBC(*10^9^/L)	8.43 ± 4.14	11.55 ± 6.49	0.049
neutrophil ratio (%)	60.1 ± 23.3	75.9 ± 12.6	0.004**
Hb(g/L)	133 ± 23	131 ± 22	0.724
Hematocrit(%)	39.7 ± 6.9	39.0 ± 6.2	0.708
PLT(*10^9^/L)	225 (188,260)	222(176,266)	0.820
ESR(mm/h)	30 (10,68)	37(26,72)	0.073
CRP(mg/L)	8.92 (3.16,79.89)	49.58(8.55,107.38)	0.122
PCT(ng/mL)	0.473(0.867,0.975)	0.163(0.087,0.689)	0.434
PT(s)	12.8(11.9,13.2)	13.6(12.1,14.4)	0.064
INR	1.15(1.10,1.21)	1.12(1.05,1.20)	0.286
PT%(%)	76 ± 18	83 ± 16	0.168
APTT(s)	34.5 ± 16.3	32.6 ± 9.0	0.615
Fib(g/L)	3.89 ± 2.42	5.06 ± 2.27	0.101
D-dimer(µg/mL)	0.40(0.25,0.98)	1.59(0.49,4.63)	0.008**
ALT(U/L)	25(12,59)	60(18,99)	0.122
AST(U/L)	31(15,53)	45(28,168)	0.070
ALP(U/L)	85(66,94)	116(72,185)	0.119
GGT(U/L)	40(25,91)	120(22,383)	0.046*
TBIL(µmol/L)	14.7(10.9,22.7)	23.0(11.3,37.0)	0.350
DBIL (µmol/L)	4.4(3.3,7.6)	4.3(0.0,10.0)	0.740
IBIL (µmol/L)	9.7(7.1,15.8)	11.0(6.4,17.0)	0.955
TG(mmol/L)	1.81(1.19,5.72)	1.81(0.83,6.42)	0.491
TC(mmol/L)	5.49(3.82,6.47)	4.80(3.69,6.64)	0.624
BUN(mmol/L)	3.47(2.91,4.68)	5.00(2.82,6.60)	0.086
Cr(µmol/L)	68 ± 30	69 ± 21	0.902
Ca^2+^(mmol/L)	2.26 ± 0.11	2.23 ± 0.17	0.484
amylase (U/L)	435(241,718)	591(394,1333)	0.044*
lipase(U/L)	988(648,1067)	1686(525,2675)	0.027*
CA 19 − 9(U/L)	19.18 ± 9.77	32.30 ± 29.79	0.104

## Discussion

**Fig. 1:**
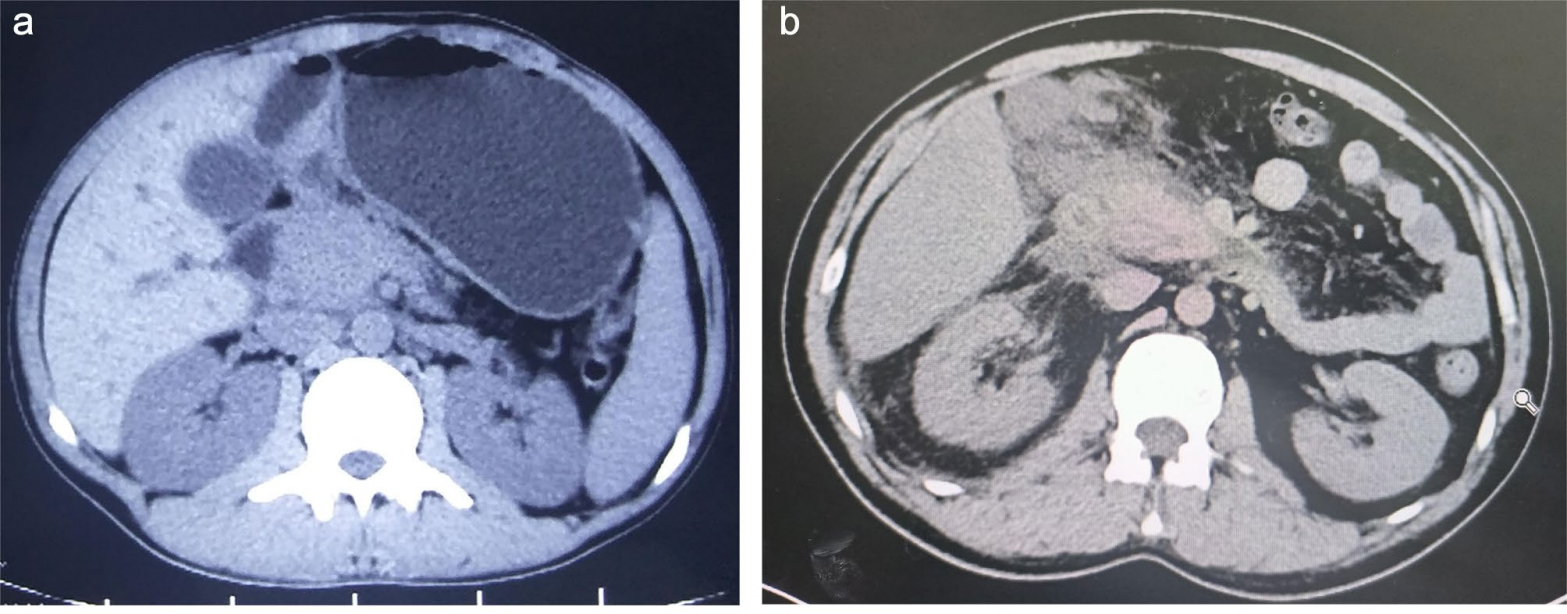
**(a)** imaging of the patient with focal AP; **(b)** imaging of the patient with non-localized AP

Focal AP is a special type of AP that radiographically present as focal inflammatory enlargement of the pancreas (Fig. 1), but for focal AP diagnosed by imaging and clinical diagnosis, are there differences in etiology composition, clinical manifestations, history, laboratory tests, compared with non-localized AP, there are few studies. Based on above questions, in this study we analyzed the differences between focal AP and non-localized AP, aim to help clinicians to identify such patients more quickly and accurately, to give timely and effective treatment interventions.The study found that changes in symptoms, signs and laboratory indicators of focal AP are not obvious, when compared with non-localized AP, however,there was no significant difference in the severity of the two groups.

A total of 51 patients were included in this part of the study, including 24 patients with focal AP and 27 patients with non-localized AP. There were no statistically significant differences in age and sex composition between two groups, indicating that the incidence of focal AP and non-localized AP was similar.

Compared with non-localized AP group, the etiological composition of focal AP group was still biliary origin first. Two cases of pancreatitis due to tumor-related factors appeared in focal AP group, both of which were confirmed by histopathology as early PDAC at the head of the pancreas, and were considered to be related to tumor invasion of the pancreatic duct, branch pancreatic duct obstruction leading to chronic obstructive pancreatitis. Although the difference in the composition ratio of this factor was not statistically significant in the two groups, but in combination with the existing literature reports of tumor-related FP [2, 14], it also suggests that we should pay more attention to the etiology screening of patients with focal AP in clinical work, and timely detection of potential malignancy.

In the study,we found that severity of the disease between two groups was with no statistically significant difference,which means despite the pancreas inflammation was localized in imaging, the condition was not be milder,patients were still at risk to become MSAP or SAP.

We found that both groups of patients presented with abdominal pain in different parts, and the location differences in abdominal pain were not statistically significant, suggesting that the location and extent of inflammation of pancreas tissue did not have a significant correlation with the location of body surface pain. However, patients with non-localized AP more likely to develop abdominal tenderness than patients with focal AP, which means symptom of focal AP patients was more atypical and the patients were more likely to be missed. Since abdominal tenderness was associated with irritation of the peritoneum by inflammation of pancreas tissue, it can be speculated that the difference in this sign between the two groups is related to the greater range of inflammation in non-localized pancreatitis.

In this study, we found that there are statistically significant differences in neutrophil ratio, D-dimer, γ-glutamyltransferase (GGT), amylase, and lipase between the two groups. The peripheral blood neutrophil ratio in non-localized AP group was significantly higher than that in focal AP group, indicating that the inflammatory response of the former was more intense and more influential than that of the latter [15, 16], at the same time, microcirculation disorders are more likely to occur, which result in the serum D-dimer difference between the two groups. The serum GGT level found in this study is higher in the non-localized AP group than the focal AP group, which may be due to the following two reasons: one is the oxidative stress response in patients with non-localized AP was more intense, resulting in higher GGT levels; the second is that non-localized AP was more likely to be combined with biliary obstruction and intrahepatic cholestasis [17–19]. In focal acute pancreatitis,there were 17 and 22 patients met the criterion(being at least 3 times above the upper reference limits) for serum amylase and lipase, in non-localized acute pancreatitis,that numbers were 17 and 23.We found that there was differences in serum amylase and lipase levels between patients in two groups, the changes of amylase and lipase in non-localized AP group were more obvious than in focal AP group, but considering that amylase and lipase did not reflect the severity of the disease [20, 21], it could not be speculated that non-localized AP was more serious than focal AP. There have been lots of study show that elevated C-reactive protein, procalcitonin, erythrocyte hematocrit, creatinine, and urea nitrogen all indicating the condition become severe [22–25], considering there were no statistically significant differences in the above indicators between the two groups, which further confirmed that there was no significant difference in the severity of the two groups.We noticed that the laboratory changes of neutrophil ratio,D-dimer, GGT, amylase, and lipase were not so much evident in focal AP, compared with non-localized AP,which might result in missing diagnosis.

The disadvantages of the study are that the sample included is small, and the larger sample size and multicenter research work are still needed in the future. This study is a retrospective study, and it failed to dynamically monitor the changes of some index values, and did not conduct regular follow-up to explore the disease outcome, which may make the research results less accurate.

## Conclusion

Compared with patients with non-localized AP, patients with focal AP have a smaller proportion of bloating and abdominal tenderness, and the levels of neutrophil ratio, D-dimer, GGT, amylase, and lipase are lower. However, there was no significant difference in the severity between two groups of patients. Therefore, although focal AP is a limited inflammatory change in imaging, it does not show a milder disease than non-localized AP, on the contrary, focal AP is clinically more difficult to diagnose in a timely and accurate manner due to the insignificance of symptoms and changes in certain laboratory indicators, which requires the attention of clinicians.

## Data Availability

The datasets used and analyzed during the current study are available from the corresponding author on reasonable request.

## Abbreviations

FP: Focal pancreatitis
AP: Acute pancreatitis
CT: Computed tomography
MSAP: Moderately severe acute pancreatitis
SAP: Severe acute pancreatitis
CP: Chronic pancreatitis
WBC: White blood cell count
Hb: Hemoglobin
PLT: Platelet count
ESR: Erythrocyte sedimentation rate
CRP: C-reactive protein
PCT: Procalcitonin
INR: International normalized ratio
PT: Prothrombin time
APTT: Activated partial thromboplastin time
Fib: Fibrinogen
ALT: Alanine aminotransferase
AST: Aspartate aminotransferase
ALP: Alkaline phosphatase
GGT: γ-glutamyltransferase
TBIL: Total bilirubin
DBIL: Direct bilirubin
IBIL: Indirect billirubin
TG: Triglyceride
TC: Total cholesterol
BNU: Blood urea nitrogen
Cr: Creatinine
PDAC: Pancreatic ductal adenocarcinoma
